# Contact heat thermal threshold testing in beagle dogs: baseline reproducibility and the effect of acepromazine, levomethadone and fenpipramide

**DOI:** 10.1186/1746-6148-8-206

**Published:** 2012-10-30

**Authors:** Marina Verena Hoffmann, Sabine Beate Rita Kästner, Manfred Kietzmann, Sabine Kramer

**Affiliations:** 1 Department of Small Animal Medicine and Surgery (Hoffmann, Kästner and Kramer), Bünteweg 9, D-30559, Hannover, Germany; 2 Department of Pharmacology and Toxicology (Kietzmann), University of Veterinary Medicine Hannover, Bünteweg 17, D-30559, Hannover, Germany

**Keywords:** Thermal threshold, Levomethadone, Acepromazine, Plasma concentration, Reproducibility, Dogs

## Abstract

**Background:**

In this methodology article a thermal threshold testing device designed to test nociception in cats was assessed in six dogs. The purpose of this study was to investigate baseline reproducibility of thermal thresholds obtained by the contact heat testing device, to assess the influence of acepromazine and levomethadone and fenpipramide in dogs. The relationship between change in nociceptive thermal threshold and the opioid′s plasma concentration was determined. Six adult beagle dogs received levomethadone (0.2 mg/kg), acepromazine (0.02 mg/kg) or saline placebo by intramuscular injection (IM) in a randomized cross-over design. Three baseline nociceptive thermal threshold readings were taken at 15 minutes intervals prior to treatment. Further readings were made at 15, 30, 45, 60, 90, 120, 150, 180, 210, 240, 270, 300, 330, 360, 420 and 480 minutes after injection. A sedation score was assigned at every reading. Four saline placebo treatments were performed to assess baseline reproducibility. Levomethadone serum concentrations were measured prior and 0.5, 1, 2, 4, 8, 12 and 24 hours after drug dosing in a separate occasion.

**Results:**

Acepromazine did not seem to increase the thermal threshold at any time. After levomethadone there was a significant rise of the thermal threshold between 15 to 120 minutes at serum concentrations between 22.6-46.3 ng/mL. Baseline reproducibility was stable in adult beagle dogs.

**Conclusion:**

The thermal threshold testing system is a suitable device for nociceptive threshold testing in dogs.

## Background

Prior to clinical use of analgesic drugs in animals, objective analgesiometric studies are necessary to assess the efficacy of the analgesic drugs. The purpose of this study was to adapt a nociceptive thermal threshold testing device developed for the use in cats [1] to dogs, to evaluate baseline reproducibility of thermal thresholds in adult beagle dogs and to investigate the influence of sedation and opioid administration. The relationship between thermal nociceptive thresholds and plasma concentrations of levomethadone were determined. Our hypothesis was that the μ-agonist levomethadone will induce an increase in superficial thermal thresholds.

## Methods

### Dogs

Six healthy purpose-bred beagle dogs (2 spayed females, 4 castrated males) aged 6–8 years, with a mean weight of 17.8 kg (± 2.0) were studied. They were housed in groups and fed a commercial moist whole diet (Hills Canine P/D Prescription Diet™) twice a day. The dogs were habituated to wearing the nociceptive thermal threshold (TT) testing devices and to the area where the testing was performed. The study was approved by ethical review (licensed by the Lower Saxony State Office for Consumer Protection and Food Safety, 33.9-42502-04-07/1417) according to regulations of the German Animal Welfare Act.

### Nociceptive thermal threshold testing

The thermode based nociceptive thermal threshold testing device for ramped heating used in this study has previously been described [1]. The equipment was developed for the use in cats and adapted for the use in dogs. The thermal probe remained unchanged and only the dimensions of the elastic bands used to attach the probe to the thorax of the dogs were modified. Briefly, a thermal probe acting as a temperature sensor and heater element was attached to the dogs′ clipped thoracic wall by an elastic band in the centre of an inflatable bladder to provide constant contact between the probe and the skin. The skin temperature was recorded and the probe was heated at a constant rate of 0.6°C per second until the dog reacted to the thermal stimulus or the safety cut-out of 55°C was reached. All assessments were made by the same observer who was blinded to treatment. A skin flinch and/or a head turn towards the probe, was considered a positive reaction. At this point the heating was stopped immediately and the threshold temperature recorded. All dogs tolerated the equipment well and the cable between the thermal probe and the control unit could remain attached to the thermal probe throughout a testing day. This reduced direct manipulation of the animals to a minimum.

### Instrumentation

The evening before a testing day a 12 × 10 cm square on the dogs’ lateral thorax was clipped. On a testing day two dogs were placed in an area with a blanket, toys and drinking water ad libitum. The thermal probe was attached to the pressure bladder and secured against the dog`s clipped thorax with an elastic band and allowed at least 30 minutes to reach skin temperature. The pressure was measured regularly to ensure constant contact over time.

### Study design

Three saline placebo treatment periods at least six weeks apart were performed prior to drug studies. Additionally levomethadone, acepromazine and saline placebo were assessed in a randomized cross over study.

Three baseline readings were taken at 15 minutes intervals prior to administration of levomethadone, acepromazine or saline placebo. Levomethadone is licensed for dogs in Germany as a mixed preparation in combination with the parasympatholytic compound fenpipramide, to offset opioid induced vagal tone (L-Polamivet, Hoechst AG, Frankfurt, Germany). L-Polamivet contains 2.5 mg/mL levomethadon combined with 0.125 mg/mL fenpipramide. The dogs were treated with levomethadone 0.2 mg/kg (combined with 0.01 mg/kg fenpipramide), acepromazine 0.02 mg/kg or 1 ml saline placebo intramuscularly (IM) into the semimembranous/semitendinous muscle group using a 20 G needle (Terumo Europe NV, Leuven, Belgium). Further readings were made at 15, 30, 45, 60, 90, 120, 150, 180, 210, 240, 270, 300, 330, 360, 420 and 480 minutes after injection. There was a washout of at least one week between treatments and the thorax side was changed after every treatment in a randomized cross over design.

Prior to the experiments, and 6 times during the study, the probes were calibrated against a thermometer and the results were adjusted on the basis of the derived regression.

### Reproducibility

Four saline placebo treatments were performed over a six month period to assess baseline reproducibility for the thermal testing system. The results were compared between the six dogs and the four different placebo groups.

### Sham testing

All six untreated dogs were each tested five times with the equipment randomly switched off and assessed by a blinded observer. Each test was terminated after 45 seconds if no response had occurred.

### Influence of sedation

The degree of sedation was assessed using a numeric descriptive scale (NDS). The NDS consisted of a scale ranging from 0 to 3, with 0: no sedation; 1: mild sedation (less alert but still active); 2: moderate sedation (drowsy, recumbent but can walk); and 3: intense sedation (very drowsy, unable to walk) [2].

Sedation score was assigned at every reading and the area under the sedation score (AUC) was calculated and compared between treatment groups. Acepromazine was administered to evaluate the influence of sedation.

### Blood sampling

The same 6 dogs were used for the pharmacokinetic study but TT and blood collection was performed on a separate occasion to minimize manipulation during the thermal testing and to avoid stress induced changes in the testing results. Samples were withdrawn from the cephalic vein using a 20 G needle (Terumo Europe NV, Leuven, Belgium) before and 0.5, 1, 2, 4, 8, 12 and 24 hours after drug dosing. The blood was sampled in lithium heparin tubes and centrifuged (12000 rpm) for 2 minutes. Serum was separated and stored at - 18°C for one week before levomethadone assays were performed.

### Drug analysis

Levomethadone concentrations were measured after serum samples were precipitated with zinc sulphate and methanol. Quantitative analysis was performed by HPLC-Triplequad-MS (LIPIDOMIX GmbH, Berlin, Germany). Measurements were performed in multiple reaction monitoring (MRM) mode. Two MRM transitions were used for compound identification. Methadone-D9 and EDDP-D3 were used as internal standards and calibration was performed with 8 different concentrations in dilution series of 1:2. Calculation of the calibration was performed using Agilent MassHunter Software (Agilent Technologies GmbH, Böblingen, Germany). The limit of quantification (LOQ) was 0.001 ng/ml. Calibration curve data was constructed in the range of expected concentrations of 25.0-0.20 ng/mL methadone. The regression correlation coefficient (r) of the standard curve was 0.99999.

### Pharmacokinetic analysis

The pharmacokinetic analysis was performed using WinNonlin 3.2 (Pharsight Corporation, Mountain View, California, USA). The goodness of fit for the one-compartment model used was evaluated based on the R^2^ and the values of the Akaike and Schwartz criteria. Area under the concentration-time curve (AUC) was calculated as well as the elimination half life (t1/2). The peak concentration (Cmax) and time to peak concentration (Tmax) were determined according to standard formula.

### Statistical analysis

Statistical analysis was carried out using SigmaStat® version 3.5 and SAS Version 9.0. The graphic presentation was done using GraphPadPrism® version 5.0.

Mean ± SD are reported unless otherwise stated. The data were checked for normality using the Shapiro-Wilk test. Variables not normally distributed were analyzed by a Friedman test and a Wilcoxon signed-rank test.

The three baseline readings were averaged to give a mean ± SD baseline value. Taking this value as time 0, repeated-measures ANOVA followed by Dunnett′s test was used to assess changes with time in response to treatment. The area under the threshold curve (AUC) was calculated for the time of significant threshold rise for each drug and compared to each other as well as to saline placebo using a Wilcoxon-signed-rank test (sum of AUC). The area under the sedation curve (AUC) was calculated for the time of significant degree of sedation and compared among treatment groups using a Wilcoxon-matched pairs test (sum of AUC). Multi-Factor Analysis Variance was used to compare the interaction of the six dogs in relation to measuring time point and saline placebo period to assess reproducibility. P < 0.05 was considered significant.

## Results

**Figure 1:**
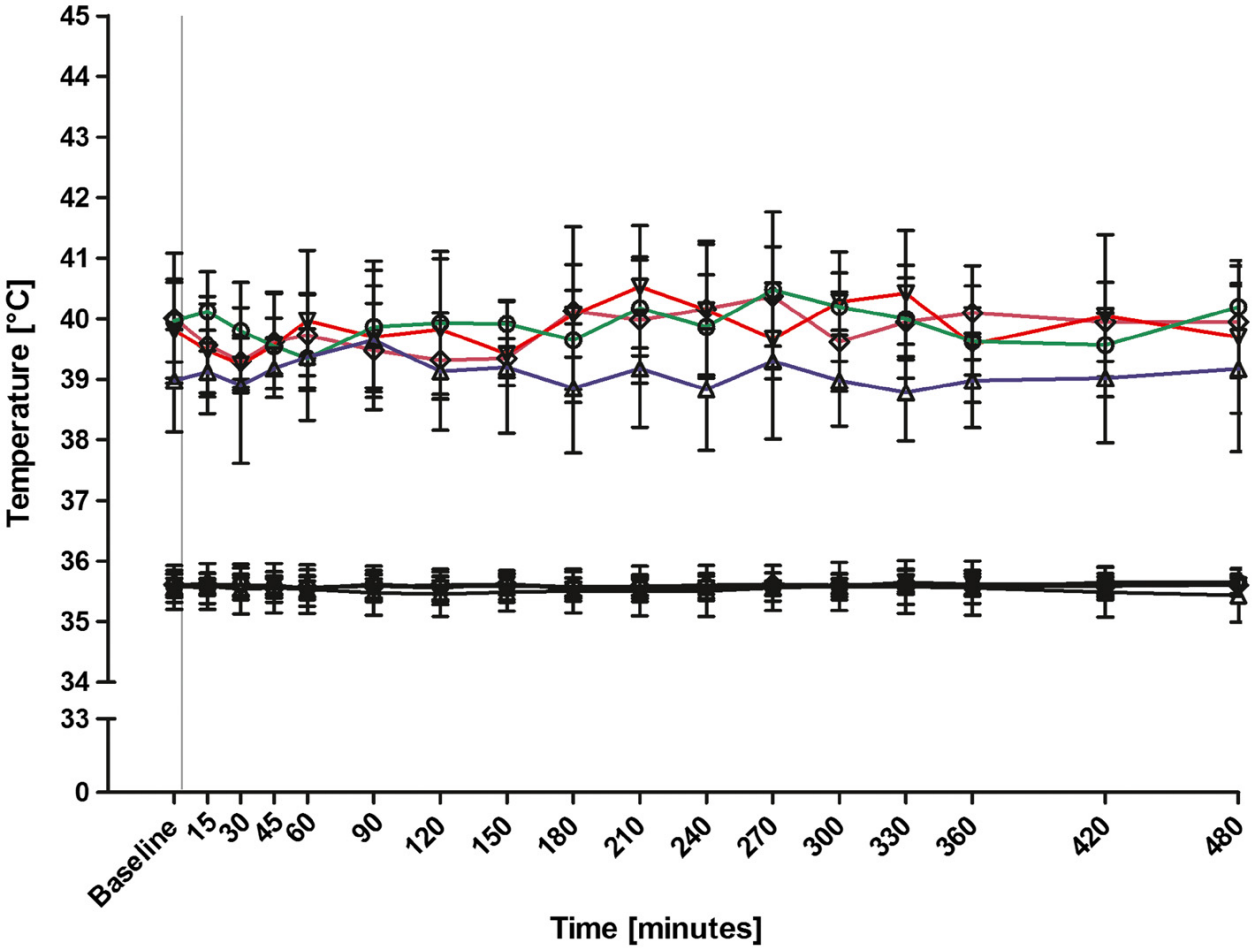
Mean ± SD thermal threshold (colored lines) and skin temperature (black lines) in °C after 4 saline treatments (gray vertical line) over a six months period in 6 dogs.

The mean nociceptive thermal threshold of all six dogs and 4 saline placebo treatments and for all time points was 39.7°C ± 0.4°C. There was no significant difference between the four saline placebo groups over the six months period (Figure 1). Sham testing gave no false positives and no false negatives. Following the intramuscular dose of 0.2 mg/kg levomethadone the mean maximum serum concentration of 46.3 ± 14.03 ng/mL was reached after 1.25 hours. The area under the curve (AUC) was 185.4 hr*ng/mL. Serum concentration decreased with a half life of 2 hours.

**Figure 2:**
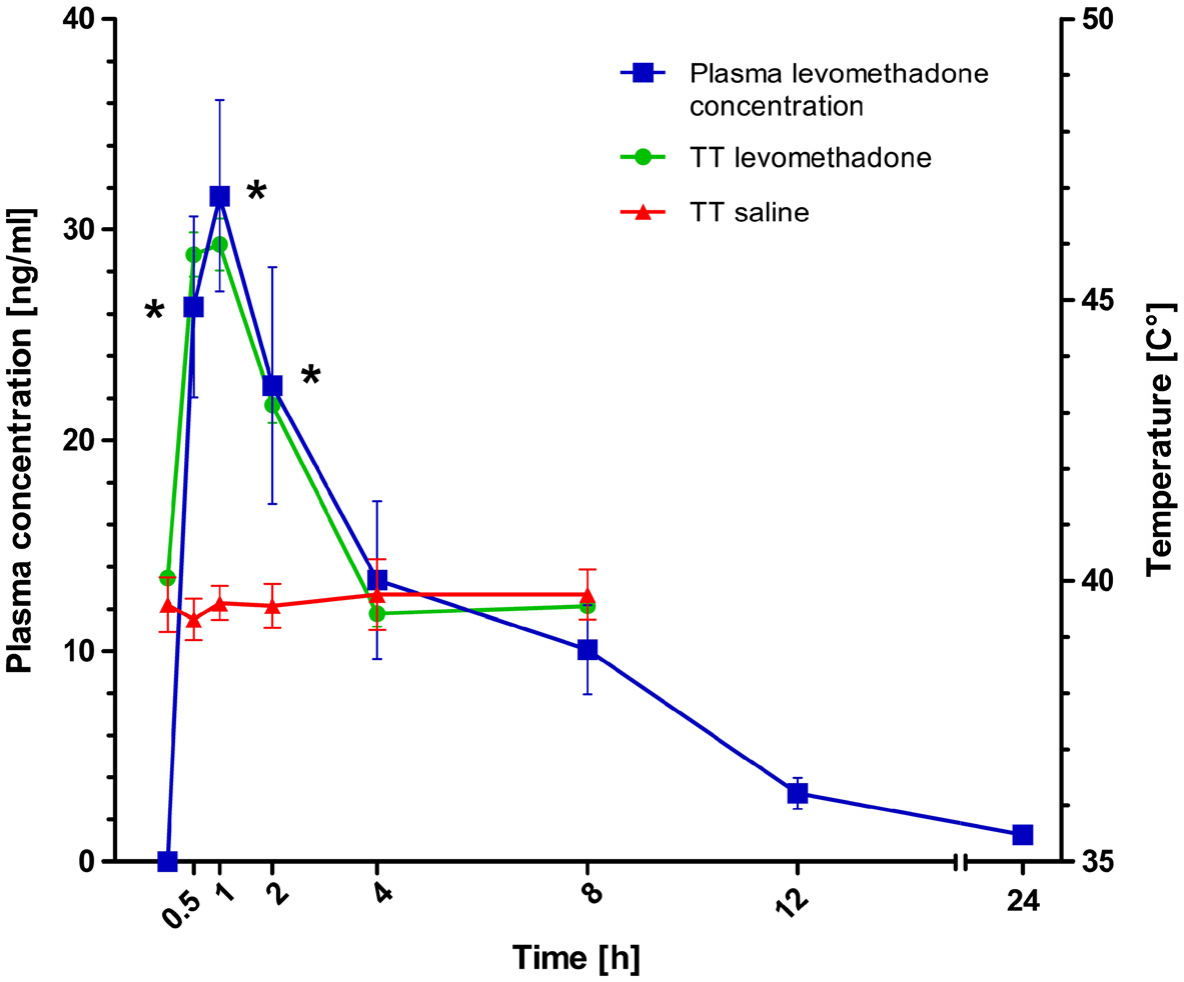
**Thermal threshold in relation to plasma concentrations.** The green graph (circles) shows the thermal threshold (°C) after administration of 0.2 mg/kg levomethadone intramuscularly. The red graph (triangles) illustrates the thermal threshold (°C) of the 4 saline treatments. The blue graph (squares) demonstrates the plasma levomethadone concentration (ng/mL). Note the time points of significant threshold rise (stars). All data are displayed as Mean ± SD of the 6 dogs.

After injection of 0.2 mg/kg levomethadone IM, there was a significant rise of the nociceptive thermal threshold between 15 to 120 minutes compared to baseline, saline placebo and acepromazine. The maximum threshold was 46.0°C ± 2.2°C 45 minutes after injection. The serum concentration during a significant threshold rise was 22.6-46.3 ng/mL (Figure 2).

Acepromazine did not significantly increase the nociceptive thermal threshold at any time (40.4°C ± 1.2°C). The dogs had a sedation score between 1 and 3, 15 to 180 minutes after treatment with a significant increase of sedation between 45 to 120 minutes (1.75 ± 0.5) compared to baseline (0).

Levomethadone led to a sedation score between 1 and 3 from 30 to 270 minutes after treatment, with a significant increase compared to baseline from 30–210 minutes (1.98 ± 0.49). There was no significant difference between the degree of sedation after administration of acepromazine compared to levomethadone (p = 0.06). Degree of sedation induced by IM acepromazine or levomethadone was significantly different to saline-placebo (p < 0.001).

## Discussion

It is well recognised that nociceptive threshold testing does not provide the same stimulus as clinical pain, but it can be used to assess the efficacy of analgesics with cutaneous antinociceptive activity in the preclinical phase for elaboration of adequate doses and dosing intervals.

In the last few decades many different techniques have been developed to perform thermal threshold testing in dogs. Both fixed stimulus, where latency to response is measured, and a threshold stimulus that elicits an effect have been used as end points in analgesiometry. In 1941 Andrews and Workman [3] used a “Hardy-Wolff” apparatus [4] for thermal threshold testing in dogs. They blackened the dogs` thoracolumbar area of the skin and stimulated with radiant heat. In another study, a filament lamp was attached to the hindlimb of dogs for thermal threshold testing [5]. In a recent study, latency response was measured following application of a relatively constant temperature of 60°C with a thermal probe [6].

In the present study, nociceptive thermal threshold was measured by adapting equipment well established for use in cats [7-12]. The testing device has previously been used for nociceptive thermal threshold measurements in horses [13,14], encouraging the adaption to another species.

The equipment was well tolerated by the beagles who wore the band for up to 10 hours without attempting to remove or play with it. In contrast to the first study of this device in cats [1] none of the dogs chewed the cable between the probe and the control unit. Thus there was no necessity to unplug the cable between tests which reduced direct manipulation on the dogs. The flexibility of the ribbon cable minimized the restriction on the dogs, keeping a possible influence of restraint induced stress low.

It is important to evaluate and adapt nociceptive threshold testing methods for each species due to species and individual variability. It is known that variations in skin thickness and blood flow [15], hair covering and epidermal pigmentation [16] as well as nociceptor distribution may influence the thermal threshold.

Previous studies in cats investigated reproducibility by 6–12 repeated tests at 5-15- minute intervals in eight untreated cats and further tests at 24 hours and repeated this on only two occasions 3–6 months later. Reproducibility was improved in cats during development of the equipment and testing procedure by careful attention to detail such as clipping, band placement and precise bladder pressure [1] and this accuracy was considered in the present study.

In the first study of this device in cats, a safety cut-out at 60°C led to minor skin lesions [1]. The safety cut-out was adapted to 55°C to prevent thermal burns in the present study.

Since there are many factors possibly influencing the nociceptive thermal threshold, baseline reproducibility is required prior to any testing of analgesic agents. In the present study, three of the four saline placebo treatments were assigned prior to drug testing. There was at least six weeks between saline placebo treatment periods to provide reproducible data over several months.

The effect of learning leading to recognition and subsequent avoidance of the stimulus can be another factor influencing the test results [17]. Sham testing showed that the dogs did not anticipate the experiment. The testing device used in this study, had the great advantage that direct manipulation of the dogs could be reduced to a minimum and no learned avoidance of the testing device occurred. In addition it is presumed that environmental factors influence the response to painful stimuli in animals [18,19]. Therefore the dogs were habituated to wearing the testing device, the room in which the testing was performed and the person performing the tests.

Comparable to the studies in cats and horses using the same testing device as in the current paper [1,8,13] thermal threshold did not change significantly after placebo saline injection in the present study.

Measurement of the plasma concentration of levomethadone gave us the opportunity to elucidate the relationship between blood concentration and effect (Figure 2); however, it is important to take into consideration that the analgesic effect correlates with the drug concentration at the site of action- effect site (opioid receptors in the central nervous system) and not necessarily directly with the plasma concentration. Additionally, the sampling schedule may not allow very accurate estimates of pharmacokinetic parameters. Nevertheless, a close relationship between serum concentration and nociceptive thermal threshold was demonstrated for levomethadone in this study (Figure 2).

To our knowledge there is little information about the correlation between levomethadone plasma concentration and an analgesic effect in dogs. In this study, serum concentrations of 22.6 to 46.3 ng/mL provided analgesia in dogs. These concentrations were observed during significant elevation of TT and not at steady-state, and the relationship between concentration and effect is unlikely to be direct. It is therefore likely that the changes in effect lagged behind the changes in concentration, and the actual analgesic concentrations may be therefore have been somewhat different. The duration of effect was 15 to 120 minutes after IM injection, with a maximum threshold 45 minutes after injection.

Time to maximal plasma concentration in the present study was reached after 1.25 hours, with a maximal plasma concentration of 46.3 ± 14.0 ng/ml. In a recent study, time to maximal plasma concentration was reached after 1.26 hour, with maximal plasma concentrations of 23.9 ± 14.4 ng/ml after subcutanoues administration of 0.4 mg/kg methadone hydrochloride in dogs [20]. Racemic methadone and levomethadone boths provide a comparable analgesic effect. Another study compared analgesia following administration of either 0.3 mg/kg levomethadone or 0.6 mg/kg racemic methadone intramusculary (IM) to cats undergoing ovariohysterectomy. Post operative pain was scored at a number of time points for up to four hours after surgery using a scoring system comprised of physical and behavioural measures, including wound palpation. The authors concluded that both levomethadone and racemic methadone, at the doses tested, provided adequate analgesia after ovariohysterectomy [21].

Opioids commonly produce sedation [22] due to their interaction with μ and κ receptors [23]. Acepromazine was used to determine the influence of sedation on the testing results. It is the most widely used phenothiazine sedative in veterinary medicine, and is considered not to have a significant analgesic effect [5], although there is controversy if phenothiazines produce analgesia [24-26]. In a recent study on cats, acepromazine alone and in combination with tramadol increased the pressure threshold whereas tramadol alone did not [26]. In contrast, no increase in thermal threshold occurred after administration of acepromazine in horses, suggesting that acepromazine does not have somatic antinociceptive effects in the horse [14].

In the present study, degree of sedation was compared between treatment groups. Acepromazine produced sedation comparable to levomethadone at the dosage used, but did not increase the nociceptive thermal threshold, indicating that the testing system seems to measure only the analgesic effect and is not influenced by sedation. However, the study design and the small number of dogs tested do not seem appropriate to demonstrate the lack of an effect. Therefore, we cannot confirm with acceptable certainty that saline placebo and acepromazine do not have an effect on nociceptive thermal threshold, even though the data itself shows relatively convincingly that the thermal threshold likely does not change following saline placebo or acepromazine administration.

## Conclusion

The testing device is suitable to assess nociceptive thermal thresholds in dogs and the analgesic response to an opioid. Saline placebo thermal thresholds were stable over a six month period and testing results appeared not to be influenced by sedation alone.

Furthermore the methodology seems well suited for investigation the thermal antinociceptive profile of opioids in dogs with respect to onset and duration of effect, and concurrent blood analysis can be performed to elucidate the relationship between plasma concentration and effect, as in the current study.

## Abbreviations

TT: Thermal threshold; IM: Intramuscularly; EDDP: (2-Ethylidin-1,5-dimethyl-3,3-diphenylpyrrolidin); AUC: Area under the concentration-time curve; t1/2: Terminal half life; Cmax: Peak concentration; Tmax: Time to peak concentration; rpm: Rounds per minute; LOQ: Limit of quantification; NDS: Numeric descriptive scale.

## Competing interests

The authors declare that they have no competing interests.

## Authors’ contributions

MVH performed the TT testing, participated in the statistical and pharmacokinetic analysis and drafted the manuscript. SBRK participated in the study design and helped to draft the manuscript. MK carried out the pharmacokinetic analysis of the study and helped with the statistical analysis. SK participated in the design of the study, performed the drug administration and helped to draft the manuscript. All authors read and approved the final manuscript.
